# Dry Eye Disease in Patients with Functioning Filtering Blebs after Trabeculectomy

**DOI:** 10.1371/journal.pone.0152696

**Published:** 2016-03-31

**Authors:** Hong Ji, Yingting Zhu, Yingying Zhang, Zuohong Li, Jian Ge, Yehong Zhuo

**Affiliations:** State Key Laboratory of Ophthalmology, Zhongshan Ophthalmic Center, Sun Yat-sen University, Guangzhou, People’s Republic of China; Save Sight Institute, AUSTRALIA

## Abstract

**Purpose:**

The aim of this study was to analyze dry eye disease (DED) in patients with functioning filtering blebs and to explore the relationship between the morphology of filtering blebs and ocular surface instability.

**Methods:**

This was a cross-sectional, case-comparison study. Seventy glaucomatous patients (70 eyes) with functioning blebs who had undergone trabeculectomy more than 6 months prior (study group) and 35 control subjects (35 eyes) (control group) were included. All subjects completed an ocular symptom questionnaire that referred to the Shihpai Eye Study. Evaluation of meibomian gland obstruction, a tear film break-up time test (TFBUT), fluorescein corneal staining and a Schirmer’s tear test were then performed. Filtering bleb morphology was analyzed using Wuerzburg bleb classification scoring criteria in the study group. The presence of DED was defined as the concomitant presence of TFBUT <10 seconds and the presence of superficial punctate keratitis.

**Results:**

The patients with functioning blebs presented higher corneal staining scores (*P* = 0.012) and lower TFBUT values (*P* = 0.043) than the control group. DED was present in 28/70 patients in the study group and 6/35 patients in the control group (*P* = 0.018). More patients in the study group complained of dryness (*P* = 0.001), a gritty or sandy sensation (*P* < 0.001) and redness (*P* = 0.048). In the study group, the patients with DED were significantly different from the patients without DED in both TFBUT (*P* < 0.001) and corneal staining (*P* < 0.001). More patients in the DED group were likely to report dryness (*P* = 0.013) and watery or teary eyes (*P* = 0.012). The differences in meibomian gland obstruction scores between the study and the control group, the DED and the non-DED group were not significant (*P* = 0.105 and *P* = 0.077, respectively). The values for microcysts and bleb heights were significantly higher in the DED group (*P* = 0.040 and *P* = 0.011, respectively). A Spearman’s rank correlation showed that microcysts were positively correlated with corneal staining (r = 0.270, *P* = 0.024). Bleb height was negatively correlated with TFBUT (r = -0.299, *P* = 0.012) and positively correlated with corneal staining (r = 0.275, *P* = 0.021). The relationships between DED and microcysts and between DED and bleb height were significant (r = 0.247, *P* = 0.039 and r = 0.307, *P* = 0.010, respectively).

**Conclusion:**

DED is relatively common in patients with functioning filtering blebs following trabeculectomy. In DED patients, dryness and watery are common symptoms. Microcysts and bleb height are related to ocular surface instability and DED.

## Introduction

Dry eye disease (DED) is a multifactorial disease of the tears and ocular surface that results in symptoms of discomfort, visual disturbance, and tear film instability with potential damage to the ocular surface. It is accompanied by increased osmolarity of the tear film and inflammation of the ocular surface [1]. Many factors can lead to DED, including age, hormonal changes, environmental factors, topical medications, systemic diseases and drugs, autoimmune disease, meibomian gland dysfunction and ocular surgery [2]. In recent years, the relationship between DED and ocular surgery has commanded attention. Previous studies have shown that some surgical interventions that involve the anterior segment can cause DED, such as photorefractive keratectomy (PRK), laser *in situ* keratomileusis (LASIK) and cataract surgery [3–5]. However, the relationship between DED and glaucoma surgery is seldom reported.

Glaucoma is one of the most common chronic eye diseases that can potentially result in blindness. Trabeculectomy remains the first-line surgical treatment for glaucoma. This procedure reduces intra-ocular pressure (IOP) by creating a communication between the anterior chamber and the sub-conjunctival space through which the aqueous humor can be drained. This can form an elevation that is called a filtering bleb. Budenz et al [6] reported that compared with eyes without filtering blebs, the eyes with glaucoma filtering blebs experienced more dysesthesia meaning “a condition in which a disagreeable sensation is produced by ordinary stimulus”[7] which included pain, discomfort, burning, foreign body sensation or tearing. However, DED in patients who have filtering blebs following trabeculectomy and its relevant factors have not been well studied. This study evaluated DED in patients with functioning filtering blebs and analyzed the associations between the morphology of filtering blebs and ocular surface changes.

## Materials and Methods

### Design of the study and Participants

This was a hospital-based, cross-sectional, case-comparison and observational study. It was carried out at the Zhongshan Ophthalmic Center (Sun-Yat Sen University, Guangzhou, China) with the approval of the Ethics committee of the Zhongshan Ophthalmic Center (the methods section in this study were consistent with the study protocol which the ethics committee approved before the trial began). The IRB number is 2015MEKY076 (The date at which the ethics committee approved the study as well as the complete date range for patient recruitment: 2015.07.13–2015.08.15). The study was registered at www.chictr.org.cn (The authors confirm that all ongoing and related trials for this drug/intervention are registered) with the registration number ChiCTR-OOC-15006954. The study was conducted in accordance with the Declaration of Helsinki. Written informed consent was obtained from each subject.

Seventy primary glaucoma patients (70 eyes) over the age of 18 who had undergone trabeculectomy at least 6 months prior to their entry (between February 2003 and January 2015) to eliminate the influence of mitomycin C and who presented functional filtering blebs were analyzed in this study. Diagnoses of various subtypes of primary glaucoma were performed using the International Society of Geographical and Epidemiological Ophthalmology (ISGEO) classification scheme [8]. The criteria used to identify functional filtering blebs met the standards of successful trabeculectomy (IOP ≤ 21 mmHg without the use of glaucoma medications) [9]. Patients with IOP > 21 mmHg, secondary glaucoma, ocular surface disease prior to glaucoma surgery, multiple glaucoma surgeries, any other ocular surgery, a systemic disease that could have changed the condition of the ocular surface, or any topical medication in the studied eye during the 3 months prior to their entry into the study were excluded.

Thirty-five control subjects (35 eyes) were concurrently recruited during a similar time frame in the same institution. Patients with prior ocular surgical interventions, ocular surface disease, ocular trauma, contact lenses, administration of any topical medication in the eyes during the 3 months prior to their entry into the study, or any systemic condition or systemic medication that could interfere with the status of the ocular surface were excluded from the study.

### Ocular symptom questionnaire

All the participants were asked to complete an ocular symptom questionnaire that referred to the Shihpai Eye Study [10]. Two main factors explain our decision. First, most ocular symptom questionnaires were designed for white subjects. And because all of the subjects included in this study were Chinese and most glaucoma patients are elderly, we chose a questionnaire that was suitable for this specific population. Second, observation of various ocular symptoms and their frequencies in postoperative glaucoma patients was one purpose of our study. The Ocular Surface Disease Index (OSDI), for example, includes 12 questions. Among these, 6 questions concerning visual function are unfit for glaucoma patients due to their poor vision. Moreover, 3 of the ocular symptoms questions are not sufficiently comprehensive. The methods of this questionnaire were similar to those of the Salisbury Eye Evaluation Questionnaire (SEEQ). The symptoms include dryness, a gritty or sandy sensation, a burning sensation, sticky, watery or teary eyes, redness, crusting or discharge on the eyelashes, and itching. All of these could be answered as ‘never’, ‘rarely’, ‘sometimes’, ‘often’ or ‘always’, which corresponded to 0, 1, 2, 3, and 4, respectively [11].

### Ocular examination

After completing the questionnaire, the following tests were performed in qualified patients: an assessment of the morphology of filtering blebs and meibomian gland obstruction; a tear film break-up time (TFBUT) test, fluorescein corneal staining (punctate keratitis), a Schirmer’s tear test and an IOP measurement using Goldmann applanation tonometry. The examiner was masked to the results of the ocular symptom questionnaire.

#### Evaluation of filtering bleb morphology

The descriptions of bleb morphologies were made according to the Wuerzburg bleb classification score (WBCS) criteria [12, 13], which includes five items: vascularization, corkscrew vessels, encapsulation, microcysts and bleb height (Table 1).

**Table 1:** Parameters and scoring of the WBCS*.

Parameters	Scoring
Vascularity	3 = avascular
The parameters were scored from 0 to 3 according to the number of blood vessels in the filtering bleb.	2 = similar to adjacent conjunctiva
	1 = increased
Conjunctival vessels outside the bleb area were used as the reference criterion.	0 = massive
Corkscrew vessels	3 = none
The bleb was divided into three regions, and the parameters were scored from 0 to 3 on the basis of the extent of corkscrew vessel involvement in bleb.	2 = in one third
	1 = in two thirds
	0 = entire bleb
Encapsulation	3 = none
The bleb was divided into three regions, and the parameters were scored from 0 to 3 on the basis of the extent of bleb encapsulation.	2 = in one third
	1 = in two thirds
	0 = entire bleb
Microcysts	3 = entire bleb
The bleb was divided into three regions according to their relationship with the scleral flap, and the parameters were scored from 0 to 3 according to the bleb area that contained microcysts.	2 = lateral or medial of the flap
	1 = over the scleral flap
	0 = none
Bleb height: multiples of corneal thickness	
Observations were performed using a slit-lamp under high magnification. The bleb height was based on the highest point from the scleral surface to the bleb and the relationship between bleb height and corneal thickness (how many fold higher the bleb height was compared to the corneal thickness) was calculated.	

* Table from: Klink T, Schrey S, Elsesser U, Klink J, Schlunck G, Grehn F. [31] Interobserver variability of the Wurzburg bleb classification score. Ophthalmologica. 2008; 222: 408–413.

#### Meibomian gland obstruction

Meibomian gland obstruction was graded on a 0–3 scale on which 0 = no obstruction, 1 = plugging with translucent serous fluid when compressing the lid margin, 2 = plugging with a viscous or waxy white secretion when compressing the lid margin, and 3 = plugging with no secretion when compressing the lid margin [14, 15].

#### Tear film break-up time test

A drop of 2% fluorescein was instilled onto the inferior bulbar conjunctiva, and the patient was then instructed to blink naturally several times without squeezing to distribute the fluorescein. The time required for the first black spot or line to appear after a complete blink was determined using a cobalt blue filter on a slit-lamp biomicroscope. The duration was measured three times and averaged. TFBUT values less than 10 seconds were considered abnormal [16, 17].

#### Fluorescein corneal staining

Fluorescein staining of the cornea was graded according on a 4-degree staining scale by determining the area covered by the lesion [18] as follows: 0 = no staining, 1 = mild (a few stained punctae but less than 10% coverage), 2 = moderate (10%-50% coverage of the corneal surface), and 3 = severe (more than 50% coverage of the corneal surface). The presence of superficial punctate keratitis was defined as more than one dot of fluorescent staining on the corneal surface [2].

#### Schirmer’s tear test

A drop of 0.5% proparacaine was instilled, and any visible fluid in the inferior fornix or lid margin was gently dried using a cotton swab. A Schirmer tear filter strip (Tianjin Jingming New Technological Development Co, Ltd, China) was then placed in the lateral lower conjunctival sac for 5 min. Measurements were recorded after 5 minutes (closed eyes). A clinically abnormal Schirmer’s test was defined as ≤ 5 mm in 5 min [1, 15].

### DED diagnosis criteria

According to the Dry Eye WorkShop (DEWS) definition [1] and the DEWS diagnostic methodology of DED [19], the important factors in DED include symptoms (ocular and visual), tear film instability and ocular surface damage. In our study, the presence of DED was defined as the concomitant presence of a TFBUT < 10 seconds (indicating tear film instability) and the presence of superficial punctate keratitis (indicating ocular surface damage) [20]. The symptoms were not indispensable. There are two reasons that explain our decision to use this criteria. The first is that some post-trabeculectomy patients exhibited tear film instability and ocular surface damage but not ocular symptoms. In these patients, a DED diagnosis should be established (discordance between DED signs and symptoms)[21]. The other reason is that visual symptoms are important manifestations of DED and glaucoma, and it is therefore difficult to differentiate if they are caused by glaucoma, DED alone, or both.

### Statistical analysis

The Kolmogorov-Smirnov test was used to test the distribution of variables. Normally distributed variables were described according to the mean and standard deviation, while non-normally distributed variables were described as the median or interquartile range (IQR), and qualitative variables were described as a proportion. For group comparisons, parametric (Student’s *t*-tests) or nonparametric (Mann–Whitney tests) tests were used according to the distribution of the data for continuous variables, and the Pearson’s *χ*^2^ test was used for categorical variables. Correlations between bleb morphology and ocular signs were assessed using the Spearman´s rank correlation. Statistical analyses were performed using SPSS 17.0 (SPSS Inc., Chicago, IL, USA), and statistical significance was accepted at P < 0.05. Eyes with more clinically significant ocular signs were considered for statistical analysis.

## Results

### Demographic and clinical characteristics of participants

The proportions of male/female participants were 39/31 and 16/19 in the study and control groups, respectively, and there was no significant difference between the groups (*P* = 0.333). The ages of individuals in the glaucoma group (n = 70) and the control group (n = 35) were 53.37 ± 18.68 (18–80 years) and 55.80 ± 16.95 (25–84 years) (*P* = 0.519), respectively. In the study group, 36 patients suffered from primary open angle glaucoma, 23 had chronic angle closure glaucoma and 11 had primary acute angle closure glaucoma. In the control group, 13 subjects presented with cataracts, 13 showed a refractive error and 9 were normal (Table 2).

**Table 2:** The demographic and clinical characteristics of the sample groups.

	Study group (n = 70)	Control group (n = 35)	*P*-value*
Gender			0.333
Male	39	16	
Female	31	19	
Mean age±SD	53.37±18.68	55.80±16.95	0.519
Range in years	18–80	25–84	
Primary diagnosis	POAG = 36	Cataract = 13	
	CACG = 23	Refractive error = 13	
	PACG = 11	Normal subjects = 9	

POAG, primary open-angle glaucoma; CACG, chronic angle closure glaucoma, PACG, primary acute angle closure glaucoma.

Values are shown as the mean ± SD or the number of participants.

* *P*-values were derived from Student’s t-tests or Chi-Square tests.

### Ocular surface signs and symptoms in the study and control groups

The study group had higher logMAR visual acuity values (higher values indicate worse acuity) (*P* = 0.003) and corneal staining scores (*P* = 0.012) than the control group. The TFBUT values were statistical lower in the study group than in the control group (*P* = 0.043), and DED was significantly more common (28/70) in the study group than in the control group (6/35) (*P* = 0.018) (Table 3).

**Table 3:** Ocular surface signs in the study and control groups.

Signs	Study group (n = 70)	Control group (n = 35)	*P*-value*
VA	0.4 (0.1–0.9)	0.2 (0–0.3)	0.003^Δ^
IOP, mmHg	12.68±3.87	13.64±2.99	0.204
TFBUT, s	5 (4–6.25)	6 (4–8)	0.043^Δ^
Schirmer’s tear test, mm	8.50 (4.75–17.25)	7 (5–14)	0.405
Corneal staining	0 (0–1)	0 (0–0)	0.012^Δ^
Meibomian glandobstruction	2 (1–2)	2 (1–2)	0.105
DED	28	6	0.018^Δ^

VA: LogMAR visual acuity; IOP, intraocular pressure; TFBUT, tear film break-up time test; DED, dry eye disease.

Values are shown as the mean ± SD, the median (interquartile range) or the number of participants.

* *P*-values were derived from Student’s t-tests, Mann-Whitney U-tests or Chi-Square tests.

^Δ^Statistical significance (*P* < 0.05).

The ocular surface symptoms in the study and control groups are shown in Table 4. More patients in the study group complained of dryness (*P* = 0.001), a gritty or sandy sensation (*P* < 0.001) and redness (*P* = 0.048). There were no significant differences between the two groups in the other symptoms.

**Table 4:** Ocular surface symptoms in the study and control groups.

Symptoms	Study group (n = 70)	Control group (n = 35)	*P*-value*
Dryness	2 (0–2)	0 (0–1)	0.001^Δ^
Gritty or sandy	1 (0–2)	0 (0–0)	< 0.001^Δ^
Burning sensation	0 (0–0)	0 (0–0)	0.103
Sticky	0 (0–1)	0 (0–0)	0.646
Watery or teary	0 (0–2)	0 (0–1)	0.441
Redness	0 (0–0.25)	0 (0–0)	0.048^Δ^
Crusting on eyelashes	0 (0–0)	0 (0–0)	0.097
Itching	1 (0–2)	0 (0–1)	0.061

Values are shown as the median (interquartile range).

* *P*-values were derived from Mann-Whitney U-tests.

^Δ^Statistical significance (*P* < 0.05).

### Comparison of ocular surface signs and symptoms in the study group members with and without DED

In the study group, the data related to DED diagnoses are shown in Table 5. The patients with DED were significantly different from the patients without DED in both TFBUT (*P* < 0.001) and corneal staining (*P* < 0.001).

**Table 5:** Ocular surface parameters in the DED and non-DED groups.

Parameters	DED group (n = 28)	Non-DED group (n = 42)	*P*-value*
Age, y	55.57±17.37	51.90±19.58	0.425
Male	14	25	0.432
Female	14	17	
Postoperative duration, m	37.89±20.14	38.86±29.06	0.879
VA	0.40 (0.13–0.90)	0.35 (0.08–0.80)	0.489
IOP, mmHg	11.78±3.40	13.29±4.08	0.111
TFBUT, s	4 (3.25–5)	5.5 (4.75–9)	< 0.001^Δ^
Schirmer’s tear test, mm	8 (3.25–16.75)	9.5 (5–19.25)	0.405
Corneal staining	1 (1–2)	0 (0–0)	< 0.001^Δ^
Meibomian gland obstruction	2 (2–2)	2 (1–2)	0.077
POAG	12	24	0.241
CACG	11	12	0.350
PACG	5	6	0.688

DED, dry eye disease; VA: LogMAR visual acuity; IOP, intraocular pressure; TFBUT, tear film break-up time test; POAG, primary open-angle glaucoma; CACG, chronic angle closure glaucoma, PACG, primary acute angle closure glaucoma.

Values are shown as the mean ± SD, the median (interquartile range) or the number of participants.

* *P*-values were derived from Student’s t-tests, Mann-Whitney U-tests or Chi Square tests.

^Δ^Statistical significance (*P* < 0.05).

The patients in the DED group were more likely to report dryness (*P* = 0.013) and watery or teary eyes (*P* = 0.012) (Table 6). No significant differences were identified between the groups in the other symptoms.

**Table 6:** Ocular surface symptoms in the DED and non-DED groups.

Symptoms	DED group (n = 28)	Non-DED group (n = 42)	P-value*
Dryness	2 (0–2)	0 (0–2)	0.013^Δ^
Gritty or sandy	2 (1–2)	1 (0–2)	0.086
Burning sensation	0 (0–0)	0 (0–0)	0.224
Sticky	0 (0–1.75)	0 (0–0)	0.289
Watery or teary	1 (0–2)	0 (0–1)	0.012^Δ^
Redness	0 (0–0)	0 (0–1)	0.502
Crusting on eyelashes	0 (0–0)	0 (0–1)	0.002^Δ^
Itching	1 (0–2)	0.5 (0–2)	0.615

DED, dry eye disease.

Values are shown as the median (interquartile range).

* *P*-values were derived from Mann-Whitney U-tests.

^Δ^Statistical significance (*P* < 0.05).

### Comparison of filtering bleb morphology in the study group members with and without DED

Table 7 shows the scores for filtering bleb morphology in the DED and non-DED groups. The values for microcysts and bleb height were significantly higher in the DED group (*P* = 0.040 and *P* = 0.011, respectively).

**Table 7:** Filtering bleb morphology in the DED and non-DED groups.

Parameters	DED group (n = 28)	Non-DED group (n = 42)	P-value *
Vascularity	2 (2–2)	2 (2–2)	0.156
Corkscrew vessels	3 (3–3)	3 (3–3)	0.140
Encapsulation	2 (2–3)	2 (1.75–3)	0.878
Microcysts	2 (1–2)	1 (1–2)	0.040^Δ^
Bleb height	2 (1.5–2)	1.5 (1–2)	0.011^Δ^

DED, dry eye disease.

Values are shown as the median (interquartile range).

* *P*-values were derived from Mann-Whitney U-tests.

^Δ^Statistical significance (*P* < 0.05).

### Filtering bleb morphology and ocular surface signs

Microcysts were positively correlated with corneal staining (r = 0.270, *P* = 0.024), and bleb height was negatively correlated with TFBUT (r = -0.299, *P* = 0.012) and positively correlated with corneal staining (r = 0.275, *P* = 0.021). The relationships between DED and microcysts and between DED and bleb height were significant (r = 0.247, *P* = 0.039 and r = 0.307, *P* = 0.010, respectively) (Table 8).

**Table 8:** Spearman’s correlation coefficient (*P*-value) for the correlation between filtering bleb morphology and ocular surface parameters.

	Vascularity	Corkscrew vessels	Encapsulation	Microcysts	Bleb height
TFBUT	0.157 (0.194)	0.072 (0.555)	-0.111 (0.362)	-0.196 (0.104)	-0.299 (0.012)^Δ^
Schirmer’s tear test	0.076 (0.531)	0.011 (0.931)	-0.189 (0.117)	0.015 (0.903)	0.128 (0.290)
Corneal staining	-0.219 (0.068)	-0.230 (0.056)	0.021 (0.863)	0.270 (0.024)^Δ^	0.275 (0.021)^Δ^
Meibomian gland obstruction	0.030 (0.807)	-0.006 (0.961)	0.106 (0.384)	-0.058 (0.633)	-0.131 (0.279)
DED	-0.171 (0.158)	-0.178 (0.141)	0.018 (0.879)	0.247 (0.039)^Δ^	0.307 (0.010)^Δ^

TFBUT, tear film break-up time test; DED, dry eye disease.

^Δ^Statistical significance (*P* < 0.05)

## Discussion

This study shows that DED is relatively common in patients with functioning filtering blebs following trabeculectomy. In DED patients, dryness and watery are common symptoms. Microcysts and bleb height are associated with ocular surface instability and DED.

While the fact that dryness was more common in dry eye patients is not surprising, interestingly watery or teary eyes was also prominent. Schirmer I-tests were performed with topical anesthesia, and the results were assessed in our trial. We found that the values between the study and control groups and between the DED and non-DED groups were not significantly different. However, Celso Ricardo Neves Mendes et al [22] measured these values without using anesthesia in patients who had been treated with trabeculectomy and normal volunteers, and they found that the values were higher in the former group. Schirmer I-tests performed with anesthesia measure only basal levels of secretion without reflex tears. We therefore suggest that reflex tearing may be increased in postoperative patients, leading to watery or teary symptoms. Possible reasons for this effect include bleb formation in the ocular surface and dysesthesia.

The aqueous humor can move trans-conjunctively though microcysts, which are assumed to be a positive predictive factor for further bleb efficacy [23]. However, to some degree, they may also be related to tear film instability. This study demonstrated that there were more microcysts in the DED group and that the correlation between microcysts and corneal staining scores was positive. One possible explanation for this effect is the absence of mucins, especially MUC5AC. Analysis of the histopathology of functioning blebs showed that they were irregular in thickness and contained numerous intraepithelial microcysts [24] and a decreased density of normal goblet cells [25]. Previous studies also showed that there were numerous abnormal goblet cells that contained little or none of the gel-forming mucin MUC5AC in functioning blebs. Interestingly, these abnormal goblet cells seemed to correspond to the locations of the microcysts [26, 27]. Therefore, higher number of microcysts indicates a lower level of MUC5AC. MUC5AC, which is the main mucin component in tear film [28], is secreted solely by goblet cells and is believed to play an important role in maintaining tear film stability [29]. Its reduction may therefore result in tear film abnormalities.

In our trial, there was evidence indicating that bleb height could interfere with TFBUT and corneal staining scores, which is consistent with the result in a previous study [22]. Otherwise, we also found that bleb height was higher in the DED group than in the non-DED group. It is plausible that high blebs may interfere with lid function, which could affect the distribution of tear film on the ocular surface and lead, in turn, to tear film instability and corneal epithelial defects. Apart from bleb height, a small bleb–corneal angle can also induce tear film instability via the same mechanisms [30].

Few studies have reported on meibomian gland obstruction in patients after trabeculectomy. Our results indicate that meibomian gland obstruction were not different between the study and control groups or between the DED and non-DED groups. It is likely that the formation of filtering blebs may have no distinct effect on palpebral conjunctiva or the meibomian glands. Consequently, we hypothesize that meibomian gland obstruction may not play an important role in DED in postoperative patients. However, further investigation should be performed to test this proposal.

WBCS criteria offers an objective and standardized assessment of filtering blebs after trabeculectomy [13], further, its consistent evaluation is given by satisfactory interobserver variability [31], and we therefore selected it as the method used to evaluate filtering bleb morphologies. The items that are evaluated include vascularization, corkscrew vessels, encapsulation, microcysts and bleb height. In our study, the results showed that the first three of these items might not significantly affect ocular surface functions, but more research is needed to confirm these findings. An evaluation of the extent of the blebs is not included in this criteria because this characteristic less accurately reflects bleb functions than the area of microcysts. Previous studies have shown that the extent of blebs does not interfere with tear stability [22, 30].

In our study, we chose to evaluate patients who had undergone trabeculectomy more than 6 months prior to their entry. As we know, a single dose of mitomycin C is now widely used during ocular surgery to inhibit proliferation and fibrosis. In the early stages after an operation, ocular surface functions may be affected because of the cytotoxicity of this drug. Nevertheless, at a 6 month follow-up, the use of mitomycin C did not appear to contribute significantly to surface irregularities or tear-film dysfunction [30, 32, 33]. Therefore, in order to eliminate the influence of mitomycin C, the patients who had undergone trabeculectomy at lease 6 months were recruited.

This study has several limitations. First, it is an observational, cross-sectional research study, and obtaining an accurate evaluation of the status of the ocular surface prior to surgery is difficult. There is therefore a need for a more prospective study. Second, in addition to age and gender, other factors, such as the living and working environment and hormonal changes, can affect tear film stability. It was impossible to account for all of these confounders in the analyses. Third, other useful tests can be used to comprehensively evaluate dry eye, but they were not measured in this study. For instance, tear osmolarity, blink rate and temporal lid-parallel conjunctival folds [34–36] were not used.

Our study evaluated DED in patients with functioning filtering blebs and the relationships between the morphology of filtering blebs and ocular surface changes. Based on our results, we suggest that the ocular surface and the morphology of filtering blebs should be routinely evaluated in postoperative patients. Proper treatment options can then be adopted, if necessary, to improve a patient’s quality of life.

## Supporting Information

S1 Protocol Trial study protocol—original language. (PDF)

S2 Protocol Trial study protocol—English language. (PDF)
