# Supplementary material for: Dry Eye Disease in Patients with Functioning Filtering Blebs after Trabeculectomy
Source: PLoS One. 2016 Mar 31;11(3):e0152696. doi: 10.1371/journal.pone.0152696 (PMC4816306; doi:10.1371/journal.pone.0152696)
Supplement: S1 Protocol — (PDF) [file pone.0152696.s001.pdf]

临床研究方案

项目名称：小梁切除术后功能性滤过泡与眼表疾病的关系

申 请 人：卓业鸿

科　　室：青光眼

职　　称：教授

电　　话：13352828998

邮　　箱：zhuoyh@mail.sysu.edu.cn

版 本 号：1.0

中山大学中山眼科中心

2015　年 5 月 15 日

（本模版适用于观察性研究，仅供参考）

目录

方案摘要......3

缩略语表:......3

1. 研究背景......4

2. 研究目的......4

3. 研究方法与设计......4

4. 研究对象......4

5. 观察指标......5

6. 观察指标的测量或者评价方法......6

7. 偏倚......7

8. 剔除标准或者退出标准，排除、剔除或者退出后的安排......7

9. 风险和受益......8

10. 研究流程图......8

11. 安全性监测......9

12. 试验数据处理及相关记录保存......9

13. 数据分析......9

14. 质量控制和质量保证......10

15. 伦理注意事项......10

16. 预算和保险......10

17. 计划进度......10

参考文献......11

方案摘要

小梁切除术后滤过泡的形成可以改变眼表的正常功能从而引起眼部不适，由于目前关于成功小梁切除术后眼表疾病（Ocular surface disease，OSD）方面的研究较少，本研究的目的就是探讨成功小梁切除术后 OSD 的发病情况及与功能性滤过泡形态的相关性。该试验属于单中心，观察性，横断面研究。本试验先进行预实验，收集 10 例术后组及 10 例正常组，收集的数据通过两样本均数检验估计样本量。术后组的入选标准为 18 岁及以上，术前无眼表疾病，无可引起眼表改变的眼部手术史，全身疾病及药物史，无多次青光眼手术史（<2 次），非继发性青光眼，入组前眼部用药未超过 3 月，小梁切除术后 6 月以上在未使用任何降眼压药物的情况下眼压≤21 mmHg 的患者，正常对照组的入选标准为 18 岁及以上，未行任何眼部手术，无可引起眼表改变的全身疾病及药物史，入组前 45 天内未用任何眼部药物，无眼表疾病的患者。所有的受试者在试验过程中需进行一系列眼表检查，包括裂隙灯评价眼前节，滤过泡形态及睑板腺情况，泪膜破裂时间，角膜荧光素染色，泪液分泌试验及眼压测量。同时还需要对患者进行眼表症状调查。 所有数据均采用 SPSS 17．0 统计学软件进行统计分析。根据数据分布情况分别采用 t 检验或秩和检验，分类变量采用卡方检验。相关分析采用 Spearman 相关分析。P<0．05 差异有统计学意义。

缩略语表：

OSD：（Ocular surface disease）眼表疾病

WBCS：（Wuerzburg bleb classification score）Wuerzburg 滤过泡评分

1. 研究背景

眼表疾病（Ocular surface disease, OSD）是泪液和眼球表面的多因素疾病，能引起眼部不适，视觉障碍和泪膜不稳定，可能损害眼球表面 [1]。引起 OSD 的因素众多，包括年龄，体内激素水平，环境因素，局部药物，全身疾病及药物，自身免疫，睑板腺功能障碍及眼部手术等。目前对于青光眼患者眼表疾病的研究多集中在术前药物的影响，而术后眼表疾病的研究则较少。对于青光眼最为常见且经典的手术方式是小梁切除术，通过将房水引流至 Tenon’s 囊下形成滤过泡从而达到降低眼压的目的，然而术后滤过泡的形成会引起患者眼部不适，Budenz DL 等人报道 [2] 术后滤过泡形成的患者较无滤过泡形成的患者更易出现触物感痛(dysesthesia)，即正常刺激所产生程度不一致的感觉障碍 [3]，包括眼部疼痛，不适，烧灼感，异物感以及流泪等症状。本文的目的就是探讨成功小梁切除术后 OSD 的发病情况及与功能性滤过泡的相关性。

2. 研究目的

2.1. 拟解决的科学问题：通过本研究结果，了解成功小梁切除术后OSD的发病情况及与功能性滤过泡的相关性，为临床治疗术后 OSD 提供必要的建议，同时在手术时需考虑滤过泡的某些特性可能会对眼表造成影响，更为合理的进行手术设计及规划。

2.2. 主要研究目的：术后 OSD 发病率，眼表体征及症状的观察，滤过泡不同形态特征与OSD的关系及对眼表的影响。

3．研究方法与设计

3.1 试验设计类型：横断面研究 ：

4．研究对象

受试者的选择和入组：所有受试者均为 18 岁及以上，非孕妇，精神疾病患者，本试验分为研究组及正常对照组。

4.1 研究组入选标准：术前无眼表疾病，无可引起眼表改变的眼部手术史，全身疾病及药物史，无多次青光眼手术史（<2 次），非继发性青光眼，入组前眼部用药未超过 3 月，小梁切除术后 6 月以上在未使用任何降眼压药物的情况下眼压≤21 mmHg 的患者。

正常对照组：未行任何眼部手术，无可引起眼表改变的全身疾病及药物史，入组前 45 天内未用任何眼部药物，无眼表疾病的患者。

4.2 研究组排除标准：术前存在眼表疾病及可引起眼表改变的眼部手术史，全身疾病及药物史，多次青光眼手术史（≥2 次），继发性青光眼，入组前眼部用药超过 3 月，小梁切除术后不足 6 月，术后眼压>21mmHg 或用药情况下眼压≤21 mmHg，不能耐受本试验的患者。

正常对照组排除标准：既往眼表疾病及任何眼部手术史，具有引起眼表改变的全身疾病及药物史，入组前 45 天使用任何眼部药物， 不能耐受本试验的患者。

4.3 受试者招募方法：由于小梁切除术后患者需定期进行门诊随访，且大部分患者依从性强，因此可通过门诊随访的方式进行招募，对符合条件者首先告知及阅读临床研究方案。同时与患者沟通可能出现的风险及相应处理措施，遵循自愿原则，同意入组的患者签署知情同意书，随后进行临床试验。

4.4 精确的病例诊断标准和对照选择的原理（适用于病例对照研究）：本试验中对于成功小梁切除术的定义为术后 6 月以上在未使用任何降眼压药物的情况下眼压≤21 mmHg[4]；对于 OSD 的定义为泪膜破裂时间<10 秒及角膜存在点状浅层着染[1,5]. 本试验中研究组需排除术前眼表疾病的存在以避免对术后眼表的影响，且术后 6 月以上入组是为了避免术前抗青光眼药物及手术操作或术后炎症反应等对眼表产生影响，因此在选择对照组时同样需要排除眼表疾病，且入组前 45 天内未用任何眼部药物未手术的正常人。

5 观察指标

所有的受试者在试验过程中需进行一系列眼表检查，包括视力，裂隙灯评价眼前节，滤过泡形态及睑板腺情况，泪膜破裂时间，角膜荧光素染色，泪液分泌试验及眼压测量。同时还需对患者进行眼表症状调查。

6. 观察指标的测量或者评价方法

滤过泡形态：滤过泡形态的评价遵循 Wuerzburg bleb classification score(WBCS)标准[6,7]，包括滤过泡血管，螺旋状血管，包裹，微囊肿，滤过泡高度（见表 1）。其中高度是指滤过泡最高点到巩膜表面的距离，以角膜厚度的倍数表示。

表 1 WBCS 参数及评分

参数	评分
血管	3 = 无
	2=与周边结膜充血程度相似
	1 = 加重
	0 = 大量
螺旋状血管	3 = 无
	2 = 1/3
	1 = 2/3
	0 = 全部滤过泡
包裹	3 = 无
	2 = 1/3
	1 = 2/3
	0 = 全部滤过泡
微囊肿	3 = 全部滤过泡
	2 = 巩膜瓣及其内侧或外侧
	1 = 巩膜瓣
	0 = 无
滤过泡高度以角膜厚度的倍数表示	

睑板腺功能：睑板腺阻塞分为三级：0=无阻塞；1=压迫睑缘出现半透明浆液；2=压迫睑缘出现粘性或蜡状白色分泌物；3=压迫睑缘无分泌物 [8,9]。临床上睑板腺功能障碍定义为 2-3 级 [9,10]。

泪膜破裂时间：将 2%荧光素钠滴入患者结膜囊内并嘱其眨眼数次。将裂隙灯光线调至钴蓝光下，从最后一次眨眼到出现第一个黑点或黑线的时间为泪膜破裂时间。本试验需重复测量三次并计算平均值。泪膜破裂时间小于 10 秒为异常 [11,12]。

角膜荧光染色：根据病变的范围，角膜荧光染色分为 4 级 [13]:0=无染色；1=轻度染色（少量点状着染，范围不超过 10%）；2=中度染色（10%-50%角膜着染）；3=重度染色（超过 50%角膜着染）。角膜染色存在定义为角膜表面荧光染色点超过 1 个。

Schirmer’s 试验：将 0.5%丙美卡因滴入患者结膜囊内，棉签擦拭下穹窿及睑缘处的液体。泪液试纸（天津晶明技术发展有限公司，中国）放置下穹窿结膜囊外侧 1/3 处五分钟，五分钟后记录试纸读数。泪液分泌试验异常定义为 5 分钟读数≤5 mm[14]。

眼表症状问卷调查参考 Shihpai Eye Study[14]。症状包括：干涩，异物感，烧灼感，粘，流泪，眼红，睫毛根部鳞屑及痒。其中 0, 1, 2, 3, 4 分别对应为“无”，“很少”，“有时”，“经常”，“一直”[15]。

7. 偏倚

由于对眼表产生影响的因素较多，例如年龄，性别及环境因素等，本试验中为了减少年龄，性别及环境偏倚，在选择对照组时需进行年龄及性别匹配。同时根据研究组及对照组的生活工作环境尽量排除环境较差易容易引起眼表改变的受试者。

8. 剔除标准或者退出标准，排除、剔除或者退出后的安排

在检查过程中发现存在任何影响眼表的因素，以及操作过程有任何不适且不能耐受，或者自行要求终止试验的患者需排除。排除后对于存在眼表疾病的患者可给予局部对症治疗，若患者出现不适则立刻停止操作，及时进行监护及应急诊治，必要时还可联系多科甚

至综合医院进行会诊；对于自行要求终止试验的患者仍建议进行青光眼术后常规门诊随访。

9. 风险和受益

本试验的受益大于风险。通过对患者的眼表情况进行评估将有助于对眼表疾病作出诊断，为患者的治疗提供必要的建议及为疾病的研究提供有益的信息。而本研究的风险包括操作过程中出现眼红，流泪，干涩，短暂的疼痛，极少数人会出现由荧光素钠及泪液试纸引起的眼部不适等。由于本实验属于观察性研究，且所有的眼部检查均为临床常见，方便快捷的方法，对患者造成风险的可能性非常小。

10. 研究流程图

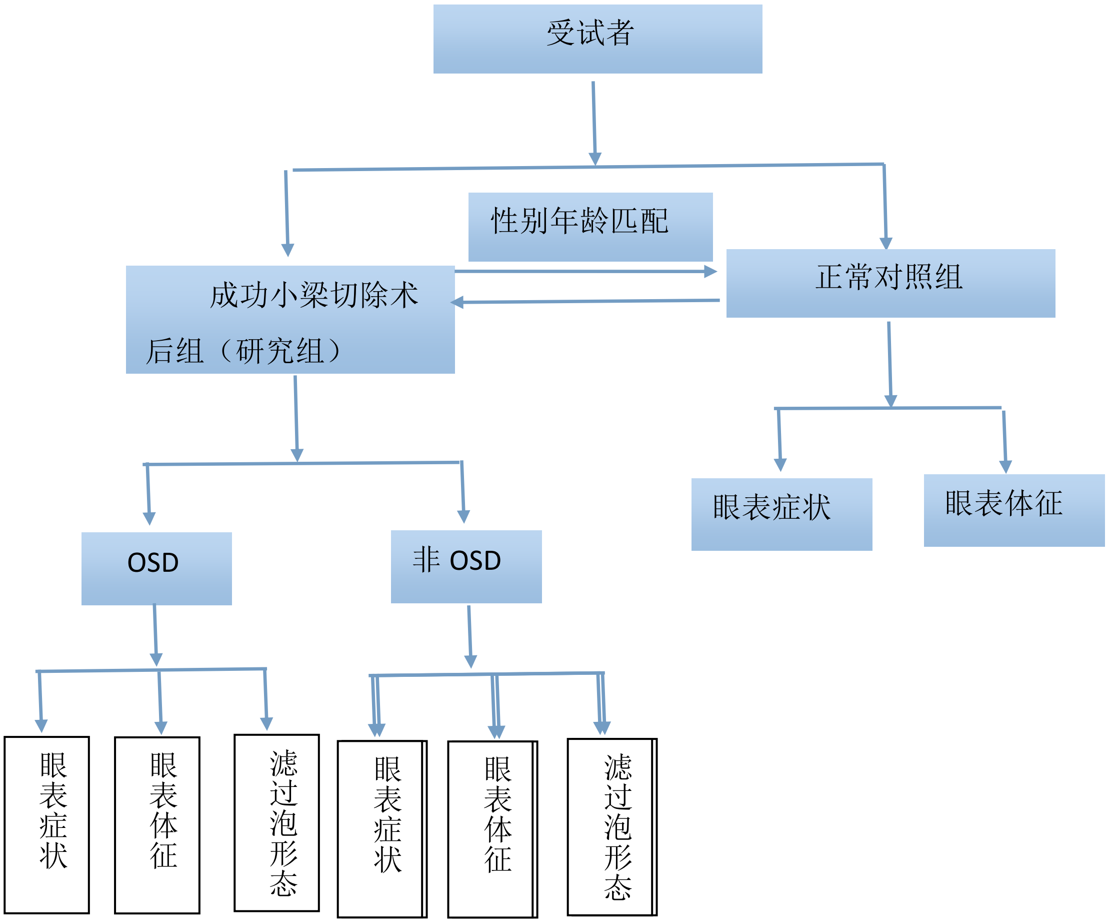

11. 安全性监测

11.1 不良事件：罕见。所有患者在临床检查过程中需严密观察患者眼部及全身情况，若出现较为明显不适，例如对荧光素钠及泪液试纸严重过敏，心率加快，低血糖等，需立刻终止检查，告知上级医师，并进行及时监护及应急诊治，必要时建立静脉通道，联系多科甚至综合医院进行会诊。

11.2 严重不良事件的报告：极为罕见。对可能出现严重不良事件，检查过程中可能出现危及生命的患者严禁入组。

12. 试验数据处理及相关记录保存

12.1 原始数据的记录和收集：研究人员在对患者检查过程中通过 Excel进行原始数据的记录和收集。

12.2 数据库管理：由参与该试验的研究人员管理。

12.3 数据保存：数据保存至研究人员的电脑。

13. 数据分析

13.1 样本量计算方法：目前类似于本试验的研究较少，较难得到总体参数，因此可行预试验来估计，预计收集 10 例研究组及 10 例正常对照组，试验设计同前，收集的数据通过两样本均数检验估计样本量。

13.2 统计分析：采用 SPSS 17．0 统计学软件进行统计分析。根据数据分布情况分别采用 t 检验或秩和检验，分类变量采用卡方检验。相关分析采用 Spearman 相关分析。P<0．05 差异有统计学意义。

13.3 要收集的数据：包括：年龄，病程，视力，眼压，BUT，基础泪液分泌值，角膜染色分级，睑板腺功能分级，滤过泡分级，眼表症状评分等。

13.4 缺失、失访以及违背方案的处理：对于缺失及违背的患者需退出本临床试验，数据不能纳入相应组。

14. 质量控制和质量保证

在试验过程中为了保证试验结果的准确性，所有的检查均需要有工作经验且操作熟练的研究人员来收集。为了避免某些结果的倾向性（例如滤过泡形态评分，BUT 及角膜染色等），眼压检查及眼表症状调查需最后进行。

15. 伦理注意事项

所有受试者均需在入组前告知试验的的基本流程，受益及风险，并签署知情同意书，在研究过程中研究者负责受试者的医疗服务，免费给受试者进行相关眼部检查并作出与临床试验相关医疗决定的措施。涉及到受试者的隐私信息需保密。对于特殊人群不进行入组。

16. 预算和保险

本试验预算包括试验材料费（300 元），资料费（200 元），查新费（500 元），论文发表费（不定）等。

17. 计划进度

**阶段目标**	**时间进度**
撰写方案和启动前准备	2015.5-2015.6
受试者入组	2015.6-2015.8
数据收集	2015.8-2015.9

注意: **如果您的项目成功入选, 在入组第一个病人前:**

1. 本项目必须获得伦理委员会的批准

2. 本项目的质量监控及数据保护方案必须获得临床研究中心的批准

参考文献

1. (No authors listed). The definition and classification of dry eye disease: report of the Definition and Classification Subcommittee of the International Dry Eye WorkShop (2007). 2007;5(2):75-92.

2. Budenz DL, Hoffman K, Zacchei A. Glaucoma filtering bleb dysesthesia. Am J Ophthalmol. 2001;131:626–630.

3. Stedman T. Stedman’s medical dictionary, 26th edition. Baltimore: Lippincott Williams and Wilkins, 2000:551.

4. Shigeeda T，Tomidokoro A，Chen YN，et a1．Long term follow-up of initial trabeculectomy with mitomycin C for primary open-angle glaucoma in Japanese patients[J]．J Glaucoma，2006，15(3)：195—199．

5. Korb DR. Survey of preferred tests for diagnosis of the tear film and dry eye. Cornea 2000; 19: 483-6.

6. Picht G, Grehn F: Development of the filtering bleb after trabeculectomy. Classification, histopathology, wound healing process. Ophthalmologe 1998, 95:380–387.

7. Picht G, Grehn F: Classification of filtering blebs in trabeculectomy: biomicroscopy and functionality. Curr Opin Ophthalmol 1998, 9:2–8.

8. Mathers WD, Shields WJ, Sachdev MS, Petroll WM, Jester JV. Meibomian gland dysfunction in chronic blepharitis. Cornea 1991; 10: 277–85.

9. Jie Y, Xu L, Wu YY, Jonas JB. Prevalence of dry eye among adult Chinese in the Beijing Eye Study. Eye(Lond) 2009; 23: 688–93.

10. Mathers WD, Shields WJ, Sachdev MS, Petroll WM, Jester JV. Meibomian gland morphology and tear osmolarity: changes with Accutane therapy. Cornea 1991; 10: 286–90.

11. Nichols KK, Nichols JJ, Zadnik K. Frequency of dry eye diagnostic test procedures used in various modes of ophthalmic practice. Cornea 2000; 19: 477-82.

12. Pflugfelder SC, Tseng SC, Sanabria O, et al. Evaluation of subjective assessments and objective diagnostic tests fordiagnosing tear-film disorders known to cause ocular irritation. Cornea 1998; 17: 38-56.

13. Bron AJ, Evans VE, Smith JA. Grading of corneal and conjunctival staining in the context of other dry eye tests. Cornea 2003; 22: 640-50.

14. Lin PY, Tsai SY, Cheng CY, Liu JH, Chou P, Hsu WM. Prevalence of dry eye among an elderly Chinese population in Taiwan: the Shihpai Eye Study. Ophthalmology 2003; 110: 1096–101.

15. Sudipta Ghosh MNAMS, Fleur O’Hare MPhil, Ecosse Lamoureux, et al. Prevalence of signs and symptoms of ocular surface disease in individuals treated and not treated with glaucoma medication. Clinical and Experimental Ophthalmology 2012; 40: 675–681.
