# Supplementary material for: Dry Eye Disease in Patients with Functioning Filtering Blebs after Trabeculectomy
Source: PLoS One. 2016 Mar 31;11(3):e0152696. doi: 10.1371/journal.pone.0152696 (PMC4816306; doi:10.1371/journal.pone.0152696)
Supplement: S2 Protocol — (PDF) [file pone.0152696.s002.pdf]

Clinical research plan

Project name：Ocular surface disease in patients with functioning filtering blebs after trabeculectomy

Proposer：Yehong Zhuo

Department：Glaucoma

Tital：professor

Telephone number：13352828998

E-mail：zhuoyh@mail.sysu.edu.cn

Version number:1.0

Zhongshan Ophthalmic Center, Sun Yat-sen University

2015.05.15

(The template is suitable for observational studies, only for reference)

Contents

Abstract....3

Abbreviations....4

1.Backgroud....4

2.Purpose....5

3.Methos and design....5

4.Object....5

5. Observed indexes....6

6.Outcome measures or evaluation methods....7

7.Bias....9

8.Exclusive criteria and arrangement after exclusion....9

9.Risk and benefit....9

10.Flow chart....10

11.Safety monitoring....10

12.Experimental data processing and records retention....11

13.Data analysis....11

14.Quality control and guarantee....11

15.Ethical considerations....12

16.Budget and insurance....12

17.Target advance....12

18.References....13

Abstract

The formation of bleb after trabeculectomy can interfere the normal function of ocular surface which leads to discomfort. At present, there have been few studies reporting the ocular surface disease (OSD) in patients with functioning filtering blebs after trabeculectomy. The aim of this study is to analyze the OSD in patients with functioning filtering blebs and to explore the relationship between the morphology of filtering bleb and ocular surface instability. This is a single-center, observational, cross-sectional study. First of all, the data of 10 surgery patients and 10 control subjects will be collected in pre-experiment to calculate the sample size. The inclusion criteria of study group includes patients over 18 year-old with functional filtering bleb who had trabeculectomy at least 6 months, without ocular surface disease prior to glaucoma surgery, without any ocular surgery or systemic disease may change the condition of ocular surface, first glaucoma surgery, primary glaucoma, without use of any topical medication in the studied eye 3 months prior to study entry, IOP≤21 mmHg without use any topical medication. The inclusion criteria of control group includes patients over 18 year-old, without any ocular surgery and OSD, without any systemic condition and systemic medication may interfere with ocular surface status, without use of any topical medication in eyes 45 days prior to study entry. All of the participants will be asked to complete ocular symptom questionnaire and submitted to several ocular examinations: assessment of the anterior segment, filtering bleb morphology and meibomian gland function; TFBUT test, fluorescein corneal staining (punctate keratitis); the Schirmer's tear test and IOP measurement. Statistical analysis is performed using SPSS 17.0. For group comparison, parametric (Student's t) or nonparametric (Mann–Whitney) tests are used according to data distribution for continuous variables, the Pearson's $\chi 2$ test for categorical variables. Correlation

between bleb morphology and ocular signs is assessed by means of the Spearman´s rank correlation. Statistical significance is accepted for $P < 0.05$.

Abbreviations:

OSD： Ocular surface disease

WBCS：Wuerzburg bleb classification score

1. Background

OSD is a multifactorial disease of tears and ocular surface that results in symptoms of discomfort, visual disturbance, and tear film instability with potential damage to the ocular surface[1]. There are many causes and factors leading to OSD, such as age, hormonal changes, environmental factors, topical medication, systemic diseases and drugs, autoimmune disease, meibomian gland dysfunction and ocular surgery. The current studies of OSD in glaucoma mainly focus on the impact of topical medication on preoperative patients, the studies reporting the OSD in postoperative patients are seldom. The most common and classic glaucoma surgery is trabeculectomy. To reduce the intra-ocular pressure (IOP), it creates a communication between the anterior chamber and the sub-conjunctival space, the aqueous humor can be drained to form an elevation which called filtering bleb. Budenz DL et al [2]reported that compared to eyes without filtering blebs, the eyes with glaucoma filtering blebs experienced more dysesthesia meaning "a condition in which a disagreeable sensation is produced by ordinary stimulus"[3] which include pain, discomfort, burning, foreign body sensation or tearing. The main purpose of the present study was

to evaluate the OSD in eyes with functioning filtering bleb and to analyze any correlation between the morphology of filtering bleb and ocular surface instability.

2. Purpose

2.1 Problems needed to be resolved in this study：In order to design surgery better and provide necessary treatment recommendations for OSD in postoperative patients, the prevalence of OSD in patients with functioning filtering blebs and any correlation between the morphology of filtering bleb and ocular surface changes should be evaluated.

2.2 The main research purpose: the prevalence of OSD, ocular surface symptoms and signs, the morphology of filtering bleb and its relationship with OSD.

3. Methods and Design

3.1 The type of study：cross-sectional.

4. Object

The age of the participants should be 18 years or older, pregnant woman and psychiatric patients will be excluded. This test divides into two groups: study group and control group.

4.1 Inclusion criteria of study group: patients who had trabeculectomy at least 6 months without ocular surface disease prior to glaucoma surgery; without any ocular surgery or systemic disease may change the condition of ocular surface; first glaucoma surgery; primary glaucoma; without use of any topical medication in the studied eye 3 months prior to study entry; IOP≤21 mmHg without use any topical medication.

Inclusion criteria of control group: patients without any ocular surgery and OSD; without any systemic condition and systemic medication may interfere with ocular surface status; without use of any topical medication in eyes 45 days prior to study entry.

4.2 Exclusion criteria of study group: patients with IOP >21 mmHg or IOP≤21 mmHg with use any topical medication; secondary glaucoma; ocular surface disease prior to glaucoma surgery; multiple glaucoma surgeries; any ocular surgery or systemic disease may change the condition of ocular surface; use of any topical medication in the studied eye 3 months prior to study entry and patients who cannot tolerate this test.

Exclusion criteria of control group: patients with prior ocular surgical interventions; ocular surface disease; use of any topical medication in eyes 45 days prior to study entry; any systemic condition and systemic medication may interfere with ocular surface status and patients who cannot tolerate this test.

4.3 Recruitment: Most of patients who underwent trabeculectomy are willing to be followed up by out-patient review, so it is a good way to recruit participants. Risk and corresponding management will be informed to qualified subjects. Written informed consent will be obtained from each subject before clinical trial begins.

4.4 Diagnostic criteria and the choice of control group (case-control study): The criteria of functional filtering bleb meets the standards of successful trabeculectomy (IOP≤21 mmHg without usage of glaucoma medications)[4]; The presence of DED is defined as the concomitant presence of TFBUT <10 seconds and the presence of superficial punctate keratitis[1,5]. In study group, the patients who are diagnosed with ocular surface disease prior to glaucoma surgery should be excluded to avoid an impact. We choose the patients who had the trabeculectomy at least 6 months to eliminate the influence of preoperative anti-glaucoma medicine, operation and postoperative inflammatory reaction. In control group, the patients who have ocular surface disease and use of any topical medication in eyes 45 days prior to study entry will also be excluded because they may interfere the function of ocular surface.

5. Observed indexes

All of the participants will be asked to complete ocular symptom questionnaire and submitted to several ocular examinations: assessment of the anterior segment, filtering bleb morphology and meibomian gland function; TFBUT test, fluorescein corneal staining (punctate keratitis); the Schirmer's tear test and IOP measurement.

6. Outcome measures or evaluation methods

Evaluation of filtering bleb morphology: The detail of the bleb morphology is conducted following the Wuerzburg bleb classification score (WBCS) criteria[6,7], which includes five items: vascularisation, corkscrew vessels, encapsulation, microcysts and bleb height (Table 1). The height of the bleb is noted using multiples of corneal thickness. It is based on the highest point from the scleral surface to the bleb.

Table 1. Parameters and scoring of the WBC

Parameters	scoring
Vascularity	3 = avascular<br>2 = similar to adjacent conjunctiva<br>1 = increased<br>0 = massive
Corkscrew vessels	3 = none<br>2 = in one third<br>1 = in two thirds<br>0 = entire bleb
Encapsulation	3 = none<br>2 = in one third<br>1 = in two thirds<br>0 = entire bleb
Microcysts	3 = entire bleb<br>2 = lateral or medial of the flap<br>1 = over the scleral flap<br>0 = none
Bleb height multiples of corneal thickness	

Meibomian gland: meibomian gland obstruction is graded into 0-3scales: 0= no obstruction; 1= plugging with translucent serous when compressing the lid margin; 2= plugging with viscous

or waxy white secretion when compressing the lid margin; 3= plugging with no secretion when compressing the lid margin[8,9]. Clinically significant meibomian gland dysfunction is defined as grade 2 or 3[9,10].

TFBUT test: A drop of 2% fluorescein is instilled and the patient asked to blink several times. The time required for the first black spot or line to appear after a complete blink is determined using the cobalt blue filter on the slit lamp biomicroscope. It is measured three times and averaged. TFBUT values less than 10 seconds as abnormal[11,12].

Fluorescein corneal staining: Fluorescein staining of the cornea is graded according to a 4-degree staining scale by determining the coverage of the lesion[13]: 0 = no staining; 1= mild (a few punctate of staining but less than 10% coverage; 2 = moderate (10%-50% coverage of the corneal surface), and 3 = severe (more than 50% coverage of the corneal surface). The presence of superficial punctate keratitis is defined as more than one dot of fluoresce in staining over the corneal surface.

Schirmer's test: A drop of 0.5% proparacaine is instilled and any visible fluid in the inferior fornix and lid margin is gently dried with a cotton swab. A schirmer tear filter strip (Tianjin Jingming New Technological Development Co, Ltd, China) is then placed in the lateral lower conjunctival sac for 5 min. Measurement after 5 minutes is recorded. A clinically abnormal Schirmer's test is defined as being ≤5 mm in 5 min[1,9].

The questionnaire refered to the Shihpai Eye Study[14]. The symptoms include dryness; gritty or sandy sensation; burning sensation; sticky; watery or teary; redness; crusting or discharge on eyelashes; itching. All of them are divided into 'never', 'rarely', 'sometimes', 'often' or 'always' which corresponded to 0, 1, 2, 3, 4, respectively[15].

7. Bias

Ocular surface can be affected by various factors, such as age, sex and environment. To reduce these bias, age and sex-matched control groups of subjects should be needed, at same time, participants who live in a bad working and living environment which may change the condition of ocular surface will be excluded.

8. Exclusive criteria and arrangement after exclusion

During these ocular examinations, subjects who exist any factors which may interfere with ocular surface status; may feel discomfort and cannot tolerate or demand to stop the test will be excluded. After exclusion, OSD patients will be treated. Examination will be stopped when they feel uncomfortable, electrocardiograph monitoring, emergency treatment, even consultation will be needed in some serious patients. To those participants who stop tests, clinical follow-up is also important.

9. Risk and benefit

In this trial, the benefit is believed to outweigh the risk. Evaluating the status of ocular surface will be helpful to disease diagnosis, provide necessary treatment recommendations and useful information for OSD in postoperative patients. The risks include redness, watery, dryness and temporary pain, a very small number of people will feel discomfort because of fluorescein or Schirmer tear filter strip. This is an observational study and all of the examinations are common, convenient and efficient, the possibility of risk is small.

10. Flow chart

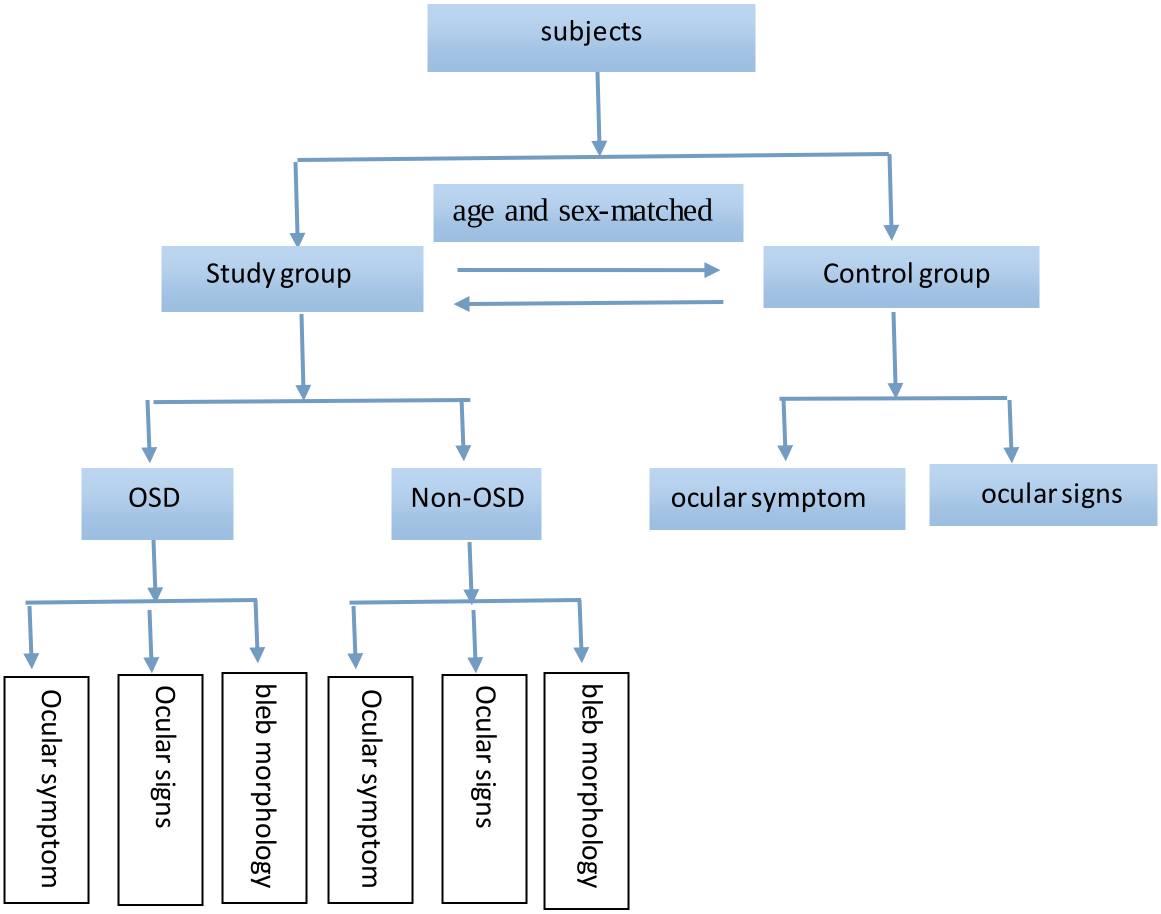

11. Safety monitoring

11.1 Adverse events: Rare. The close observation of ocular and systemic conditions is necessary. If patients feel uncomfortable, such as allergy, rapid heartbeat or hypoglycemia, the examination will be stopped immediately. In some serious patients, electrocardiograph monitoring, emergency treatment, even consultation will be needed, at same time, superior physicians must be informed.

13.2 Serious adverse events: It is very rare. Life-threatening illness is an absolute contraindication for this test.

12. Experimental data processing and records retention

12.1 Raw data record and collection: During the examination, raw data will be recorded and collected by using EXCEL software.

12.2 Database management: The researcher who take part in this study.

12.3 Data storage: The researcher's computer.

13. Data analysis

13.1 Sample size calculations: The data of 10 surgery patients and 10 control subjects will be collected in pre-experiment to calculate the sample size by using two-sample test.

13.2 Statistical analysis: SPSS 17.0 software is applied for statistical treatment. For group comparison, parametric (Student's t) or nonparametric (Mann–Whitney) tests are used, the Pearson's $\chi 2$ test for categorical variables. Spearman´s rank correlation is used for correlation analysis. Statistical significance is accepted for $P < 0.05$.

13.3 Data that needed to be collected: Age, disease course, visual activity, intraocular pressure, BUT, the Schirmer's tear test, fluorescein corneal staining, meibomian gland function, filtering bleb morphology, ocular symptom questionnaire.

13.4 The management of data missing and protocol deviation: These patients should be excluded and data cannot be included in analysis.

14. Quality control and guarantee

To guarantee the accuracy of the results, all of the examinations should be performed by skilled and expert investigators. To avoid detection bias (filtering bleb morphology, BUT and fluorescein corneal staining et al), the examiner should be masked to the results of IOP measurement and ocular symptom questionnaire.

15. Ethical considerations

All of the participants must be told the research content, benefits and risks, then sign the inform consent voluntarily. During the research process, investigators will provide trial-related medical service for free and treatment for patients if necessary. Patients´ privacy is strictly confidential. Specific populations cannot be included in this study.

16. Budget and insurance

The budget in this study includes the cost of material (300 yuan)，reference material (200 yuan)，novelty search (500 yuan), paper publishing (uncertain) et al.

17. Target advance

Stage goals	Time schedule
Proposal writing and preparation before the experiment	2015.5-2015.6
Enrollment	2015.6-2015.8
Data collection	2015.8-2015.9

Notice:

If your project is selected, the enrollment of subjects will be carry out after the following criteria is met:

1. The project must be approved by ethics committee.

2. The quality monitoring and data protection scheme of this project must be approved by clinical research center.

References

1. (No authors listed). The definition and classification of dry eye disease: report of the Definition and Classification Subcommittee of the International Dry Eye WorkShop (2007). 2007;5(2):75-92.

2. Budenz DL, Hoffman K, Zacchei A. Glaucoma filtering bleb dysesthesia. Am J Ophthalmol. 2001;131:626–630.

3. Stedman T. Stedman’s medical dictionary, 26th edition. Baltimore: Lippincott Williams and Wilkins, 2000:551.

4. Shigeeda T，Tomidokoro A，Chen YN，et a1．Long term follow-up of initial trabeculectomy with mitomycin C for primary open-angle glaucoma in Japanese patients[J]．J Glaucoma，2006，15(3)：195—199．

5. Korb DR. Survey of preferred tests for diagnosis of the tear film and dry eye. Cornea 2000; 19: 483-6.

6. Picht G, Grehn F: Development of the filtering bleb after trabeculectomy. Classification, histopathology, wound healing process. Ophthalmologe 1998, 95:380–387.

7. Picht G, Grehn F: Classification of filtering blebs in trabeculectomy: biomicroscopy and functionality. Curr Opin Ophthalmol 1998, 9:2–8.

8. Mathers WD, Shields WJ, Sachdev MS, Petroll WM, Jester JV. Meibomian gland dysfunction in chronic blepharitis. Cornea 1991; 10: 277–85.

9. Jie Y, Xu L, Wu YY, Jonas JB. Prevalence of dry eye among adult Chinese in the Beijing Eye Study. Eye(Lond) 2009; 23: 688–93.

10. Mathers WD, Shields WJ, Sachdev MS, Petroll WM, Jester JV. Meibomian gland morphology and tear osmolarity: changes with Accutane therapy. Cornea 1991; 10: 286–90.

11. Nichols KK, Nichols JJ, Zadnik K. Frequency of dry eye diagnostic test procedures used in various modes of ophthalmic practice. Cornea 2000; 19: 477-82.

12. Pflugfelder SC, Tseng SC, Sanabria O, et al. Evaluation of subjective assessments and objective diagnostic tests fordiagnosing tear-film disorders known to cause ocular irritation. Cornea 1998; 17: 38-56.

13. Bron AJ, Evans VE, Smith JA. Grading of corneal and conjunctival staining in the context of other dry eye tests. Cornea 2003; 22: 640-50.

14. Lin PY, Tsai SY, Cheng CY, Liu JH, Chou P, Hsu WM. Prevalence of dry eye among an elderly Chinese population in Taiwan: the Shihpai Eye Study. Ophthalmology 2003; 110: 1096–101.

15. Sudipta Ghosh MNAMS, Fleur O’Hare MPhil, Ecosse Lamoureux, et al. Prevalence of signs and symptoms of ocular surface disease in individuals treated and not treated with glaucoma medication. Clinical and Experimental Ophthalmology 2012; 40: 675 – 681.
